# Alcohol Consumption and Cognitive Function in an Older Population in China

**DOI:** 10.1093/geroni/igaa057.502

**Published:** 2020-12-16

**Authors:** Anying Bai, Bo Hou, Junsong Chen, Boming Fu

**Affiliations:** 1 Peking University, Beijing, China; 2 Bradford Teaching Hospitals NHS Foundation Trust, Bradford, England, United Kingdom

## Abstract

The relationship between alcohol consumption and cognitive function has only been studied to a limited extent in China. This paper examines this relationship using the China Health and Retirement Longitudinal Study (CHARLS), a nationally representative dataset of the Chinese population aged over 45. Alcohol consumption was measured by drinking status (never, former, moderate, excessive drinkers) based on number of standard drinks per week. Mental status and episodic memory function were used as measures of cognitive function. Lagged dependent variable models were used to examine independent associations between alcohol consumption and cognitive function. Our models controlled for demographic factors, socioeconomic factors, baseline cognitive functioning and an indicator for lifestyle. We also tested for an inverted J shaped relationship between alcohol consumption and cognitive functioning. A total of 10404 nondrinkers (60.09%), 2450 former drinkers (14.15%), 1599 moderate drinkers (9.24%) and 1525 excessive drinkers (8.81%) were included. Compared to never drinkers, there were no statistically significant difference between this group and moderate drinking group. While, excessive drinkers were consistently associated with on average 0.13-point decrease in episodic memory scores (p =0.031). For mental intactness, there were no statistically significant differences between never drinkers and other groups. Furthermore, we did not find evidence to support an inverted J-shaped association between standard drinks per week and measures of cognitive functioning. Excessively drinking was associated with greater decline in episodic memory function, but not in mental intactness in elder Chinese population. There is no significant association between moderate drinking and cognitive functioning measures.

